# Chemical Composition and Role of Opioidergic System in Antinociceptive Effect of *Ziziphora Clinopodioides* Essential Oil

**DOI:** 10.32598/bcn.9.5.357

**Published:** 2018-09-01

**Authors:** Faezeh Mohammadifard, Samad Alimohammadi

**Affiliations:** 1. Department of Basic Sciences, Faculty of Veterinary Medicine, Razi University, Kermanshah, Iran.

**Keywords:** *Ziziphora Clinopodioides*, Antinociceptive, Opioidergic, Rat

## Abstract

**Introduction:**

*Ziziphora Clinopodioides* has been used widely for various therapeutic purposes in Iranian folk medicine. The current study aimed to determine interaction of antinociceptive effect of the Essential Oil of *Ziziphora Clinopodioides* (EOZC) and opioidergic system in male rats using formalin test.

**Methods:**

Sixty-four male Wistar rats were divided into eight groups. The groups 1 to 7 were injected with normal saline, vehicle (Tween-80, 0.5%), 10, 20, 40 mg/kg of the EOZC, morphine (5 mg/kg) and naloxone (2 mg/kg), respectively. Thirty minutes later, the formalin test was performed by intraplantar injection of formalin (50 μL, 2%). In group 8, naloxone (2 mg/kg) was injected 15 min before injection of EOZC (20 mg/kg), followed by formalin at 15 min later. The formalin test was done as time spent for licking and biting of the injected paw. Formalin induced a biphasic pain reaction. The chemical composition of EOZC was identified using Gas Chromatography-Mass Spectrometry (GC-MS).

**Results:**

EOZC (10, 20, and 40 mg/kg) dose dependently and morphine (5 mg/kg) reduced pain responses in the both phases of pain (P<0.05). Naloxone (2 mg/kg) alone had no effect on the severity of pain (P>0.05) but pretreatment with naloxone inhibited EOZC-induced antinociception activity (P<0.05). Based on the GC-MS results, EOZC comprised 65.22% carvacrol, 19.51% thymol, 4.86% p-cymene and 4.63% γ-terpinene.

**Conclusion:**

These results demonstrate that EOZC has antinociceptive effect and this effect might mediate via opioidergic pathways.

## Highlights

- *Ziziphora clinopodioides* Essential Oil (EOZC) exhibits antinociceptive activity in the formalin test.
- Antinociceptive effect produced by EOZC is prevented by naloxone (an opioid receptor antagonist).
- EOZC seems to act via opioidergic system in the formalin test.

## Plain Language Summary

Pain is a displeasing sensation associated with tissue damage. Medicinal plants have been widely used in the treatment of pain. The essential oils are volatile molecules and have shown many biological activities, such as analgesic activity. In this study, we described the antinociceptive effect of the essential oil from *Ziziphora clinopodioides* (10, 20, and 40 mg/kg, IP). Antinociceptive activity was investigated by measuring the nociception induced by formalin. EOZC dose-dependently suppressed both phases of formalin pain. Additionally, pretreatment with naloxone reversed the antinociceptive effect of the EOZC, suggesting the involvement of opioidergic system and its receptors. These results confirm the ethnomedical uses of *Ziziphora clinopodioides*.

## 1. Introduction

Pain is a displeasing sensation associated with tissue damage (Karimi, Monajemi, & Amjad, 2014). Pain is a physiologic protective function occurring via an external or internal harmful stimulus (Ashok & Upadhyaya, 2013). Activation of nociceptors in visceral structures leads to visceral pain including angina, colic, dyspepsia, pancreatitis, appendicitis, and dysmenorrhea (de Oliveira Júnior et al., 2017). Visceral tissue injury and inflammation can activate nociceptive primary afferent fibers, which results in central sensitization or hyper-excitability of nociceptive neurons in the spinal cord dorsal horn (Grace, Hutchinson, Maier, & Watkins, 2014).

Recently, nonprescription analgesics like Non-Steroidal Anti-Inflammatory Drugs (NSAIDs) and opioids are not prescribed because of their adverse effects (Zendehdel, Torabi, & Hassanpour, 2015). Instead, a variety of plant-derived pharmaceutical products are used in traditional medicine due to their positive properties (Yama et al., 2011). Plants are a rich source of a wide variety of secondary metabolites such as flavonoids, thymol, carvacrol, terpenoids, alkaloids (Hassanpour, Sadaghian, MaheriSis, Eshratkhah, & ChaichiSemsari, 2011). Worldwide interest has increased on traditional medicine. People are more interested in consuming medicinal plants because of their therapeutic properties (Yousif, Ashoush, Donia, & Hala Goma, 2013).

Pharmaceutical research is highly focused on identifying bioactive compounds in plants. Such knowledge can be used for the treatment of different conditions, such as anxiety, pain, and inflammation (de Oliveira et al., 2012). The evaluation of pharmacological effects can be used as a strategy for discovering new plant-deprived pharmaceuticals (Zendehdel, Taati, Jadidoleslami, & Bashiri, 2011).

Medicinal and aromatic plants are traditionally used for the treatment of various illnesses (Khodaverdi-Samani, Pirbalouti, Shirmardi, & Malekpoor, 2015). *Ziziphora Clinopodioides* belongs to the Lamiaceae family. It is called “Kakuti-e-kuhi” or “Taramoshk” in Persian. This plant is spread worldwide and particularly in Iran, Afghanistan, Iraq and Turkey. Fresh leaves and stems are prescribed for wound healing and sedation. These herbs are presented in the forms of stomach tonic, antiseptic, expectorant, antifungal, antibacterial and antiseptic substances, in traditional Iranian medicine (Shahbazi, 2015).

The Essential Oil of *Ziziphora Clinopodioides* (EOZC) contains a diversity of biologically active compounds like monoterpenes and sesquiterpenes (Shahbazi, 2015). Most studies on rats and or mice with experimental models of pain have demonstrated that some of these terpenes have analgesic effects (Almeida, Navarro, & Barbosa-Filho, 2001). Therefore, the current study aimed to determine the antinociceptive effect of EOZC on opioidergic system in male rats.

## 2. Methods

### 2.1. Preparation of essential oil

Fresh leaves of *Ziziphora Clinopodioides* were collected from Gilan-e-Gharb County (Kermanshah Province, Iran) from March to July 2016. Specimen identification was demonstrated in Faculty of Agriculture, Razi University, Kermanshah, Iran. Voucher specimen (No. 6816) of the plant was deposited in the herbaria of the Research Center of Natural Resources of Tehran, Iran.

### 2.2. Isolation of essential oil

*Ziziphora Clinopodioides* leaves (100 g) were shade dried at room temperature (25±2°C). Samples were hydrodis-tilled using a Clevenger-type apparatus for 3.5 h till full of essential oil. Then, supernatant was collected and dried with 0.5 g anhydrous sodium sulfate (Merck, Darmstadt, Germany). The essential oil was stored in a dark glass bottle, and covered with aluminum foil at 4±1°C (Shahbazi, 2015).

### 2.3. Analysis of the EOZC

The chemical compounds of EOZC was determined by Gas Chromatography–Mass Spectrometry (GC-MS) (Thermo Quest Finningan, UK), as presented in Table 1. The GC-MS instrument was 5% phenyl methyl silicone and 95% dimethylpolysiloxane and equipped with DB5 capillary column (30 m, 0.25 mm, film thickness 0.25 μm). An electron ionization mode with ionization energy of 70 eV was used to determine the EOZC constituents (Shahbazi, 2015). The carrier gas was helium at a constant flow rate of 1.2 mL/min, with linear velocity of 29.6 cm/s and split ratio of 1:20. The initial oven temperature was held at 50°C for 3 min, then raised to 265°C at program ramp rate of 2.5°C/min. The final temperature was 265°C and maintained for 6°C. The temperature of the injector was 250°C. To improve accuracy of the results, the GC-MS analysis was performed in triplicate.

**Table 1.** The chemical composition of the essential oil of *Ziziphora Clinopodioides*

**Compounds**	**Composition (%)**	**Retention Time (Min)**	**Kovats Index**
Carvacrol	65.22	30.57	1315
Thymol	19.51	29.61	1293
p-Cymene	4.86	16.62	1030
γ-Terpinene	4.63	18.31	1063
E-Caryophyllene	1.07	35.47	1427
α-Terpinene	0.79	16.11	1021
Borneol	0.61	24.36	1183
Myrcene	0.51	14.62	992
Terpinene-4-ol	0.48	24.7	1190
Caryophyllene oxide	0.31	42.30	1595
α-Pinene	0.27	11.71	934
α-Thujene	0.26	11.33	927
Linalool	0.13	20.5	1105
Camphene	0.13	12.61	952
α-Phellandrene	0.13	15.58	1010
Spathulenol	0.12	42.10	1590
β-Phellandrene	0.11	16.89	1036
Limonene	0.1	16.77	1033
α-Terpineol	0.08	25.49	1206
Terpinolene	0.08	19.69	1089
1-Octen-3-ol	0.08	14.32	986
cis-Sabinene hydrate	0.07	19.02	1077
β-Pinene	0.06	14.06	981
Carvacrol, methyl ether	0.04	27.38	1246
Total	99.65		

### 2.4. Drugs

Morphine (an opioid receptor agonist), and naloxone hydrochloride (an opioid receptor antagonist) were purchased from Sigma Chemical Co. (St. Louis, MO, USA). Tween-80 and formaldehyde (37%) were purchased from Merck Co. (GERBU, Germany). All drugs were dissolved in normal saline. Various doses of the EOZC were prepared in Tween-80 (0.5%). Distilled water was used to dissolve Tween-80 into 1% (v/v) and diluted with the same volume of normal saline. The control group were injected with vehicle. All drugs were prepared before use.

### 2.5. Animals

Sixty-four male Wistar rats (weighing: 200–220 g) were obtained from the Laboratory Animal Facility of the School of Veterinary Medicine, Razi University, Kermanshah, Iran. The animals were randomly divided into 8 groups (8 in each group). Rats were maintained under standard laboratory conditions according to European Guidelines for Environmental Control in Laboratory Animal Facilities (ambient temperature of 22±1°C, 12:12 h light-dark cycle).

All animals had access to chow pellets and fresh water ad libitum. In group 1, animals were Intraperitoneally (IP) injected with normal saline, 30 minutes before intraplantar injection of formalin. In group 2, rats were IP injected with vehicle (Tween-80, 0.5%), 30 minutes before intraplantar injection of formalin. In groups 3, 4 and 5, EOZC was injected IP at doses of 10, 20 and 40 mg/kg, respectively 30 min before induction of formalin pain. In groups 6 and 7, animals received IP injection of morphine (5 mg/kg) and naloxone (2 mg/kg), respectively, 30 minutes before intraplantar injection of formalin. In group 8, animals received naloxone (2 mg/kg), then 15 min later received EOZC (20 mg/kg) followed by formalin solution after 15 min. Drug solutions were injected (1 mL/kg IP) using a 25-gauge injection needle.

To reduce the possible effect of circadian rhythm on the nociceptive susceptibility, all experiments were done from 9 AM to 12 AM (Borowicz, Kleinrok, & Czuczwar, 2003). All experimental procedures were carried out in accordance with the guidelines for the care and use of laboratory animals to investigate experimental pain in conscious animals (Zimmermann, 1983).

### 2.6. Estimation of acute toxicity

In order to identify the acute toxicity of the essential oil with few animals, a limit test was conducted according to OECD 425 guidelines. The animals were maintained in cages for at least 5 days prior to dosing to allow adaptation to the laboratory conditions. The EOZC (1000 mg/kg IP) was initially administered to one animal, followed by 24 hours observation. If the animal survived, 4 additional animals were sequentially administered with EOZC (1000 mg/kg, IP) under similar conditions. A total of 5 animals were tested. Observation was carried on for 14 days.

### 2.7. Formalin test

The formalin test is frequently used as a valid model of pain (Erami, Azhdari-Zarmehri, Imoto, & Furue, 2017). To minimize the possible effect of stress during the study, rats were placed inside a Plexiglas observation chamber (30×30×25 cm^3^) equipped with a mirror angled at 45° below the chamber for 30 minutes for 3 consecutive days (Abbott & Bonder, 1997). In the test day, a 30-minute adaptation period was applied on the animals, prior to administrating the test. Formalin (50 μL, 2%) was injected subcutaneously via a 30-gauge needle into the plantar surface of the right hind paw (Sofiabadi et al., 2014).

Following the formalin injection, rats were immediately returned to the observation chamber. The time spent on licking and biting of the injected paw was determined as nociceptive behavior. The formalin-induced behavioral responses were biphasic, as follows: 0–5 minutes (first phase, neurogenic phase) and 15–45 minutes (second phase, inflammatory phase) (Tamaddonfard & Hamzeh-Gooshchi, 2010).

### 2.8. Statistical analysis

The obtained data were prepared in Excel and Analyzed by the Analysis of Variance (ANOVA) and Tukey’s HSD post-hoc test using SPSS. The Student t test was employed to determine the differences between the 2 control groups of formalin test. The results were expressed as Mean±SEM. P<0.05 was defined to set the significant differences between the groups.

## 3. Results

### 3.1. Analysis of the EOZC

Table 1 lists the composition of the EOZC. In total, 24 components were identified, covering 99.65% of the total composition. Regarding the chemical constituents, carvacrol (65.22%), thymol (19.51%), p-cymene (4.86%) and γ-terpinene (4.63%) were the main components of the EOZC (Table 1).

### 3.2. Acute toxicity testing

Single dose acute toxicity of the EOZC was demonstrated through a limit test (1000 mg/kg. IP). EOZC caused no animal mortality in a period of 14 days. Therefore, LD50 of the EOZC was considered to be more than 1000 mg/kg.

### 3.3. Effect of the EOZC on formalin-induced pain behaviors

**Figure 1.**
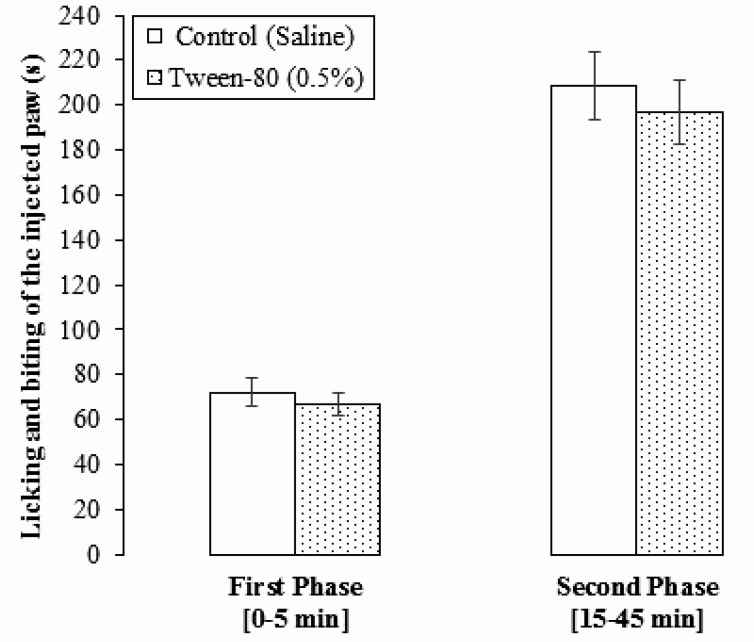
Effect of normal saline and vehicle (Tween-80, 0.5%) on the formalin-induced pain responses in rats Data are expressed as Mean±SEM (n=8).

The intraplantar injection of formalin 2% produced a biphasic pain-related behavior. Effects of normal saline and vehicle (Tween-80, 0.5%) on licking and biting time of the injected paw in male rats are presented in Figure 1. No significant differences were observed on the first phase of pain in the control (72.16±6.39 s) and Tween-80 (0.5%) (66.50±5.02 s) groups (P>0.05). In addition, no significant differences were observed on the second phase of pain in the control (208.50±15.21 s) and Tween-80 (0.5%) (196.66±14.43 s) groups (P>0.05) (Figure 1). Therefore, the data obtained from the experimental groups were compared with vehicle treated group.

**Figure 2.**
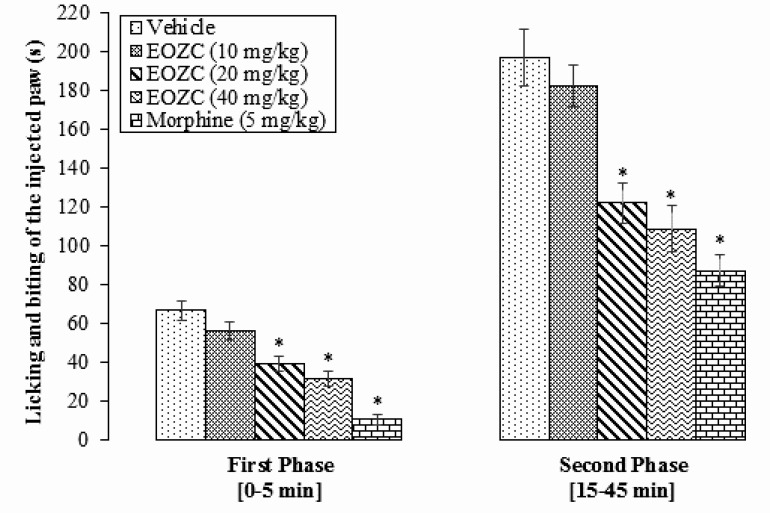
Effect of different doses of the EOZC and morphine on the formalin-induced pain responses in rats Data are expressed as mean±SEM (n=8). ^*^P<0.05: compared with vehicle test group. EOZC: Essential Oil of *Ziziphora Clinopodioides*.

The EOZC at dose of 10 mg/kg did not show any significant effect on both first and second phases of formalin pain in comparison with vehicle treated group (P>0.05). However, 20 and 40 mg/kg of EOZC induced a significant reduction in the pain response compared to the vehicle group in a dose-dependent manner in both first (39.16±3.80 s and 31.33±3.76 s, respectively) and second (121.66±10.44 s and 108.50±11.87 s, respectively) phases (P<0.05). As expected, the standard drug morphine (5 mg/kg) significantly decreased the nociceptive response in both first (10.33±2.61 s) and second (87.00±8.05 s) phases of formalin test, compared to vehicle treated group (P<0.05) (Figure 2).

**Figure 3.**
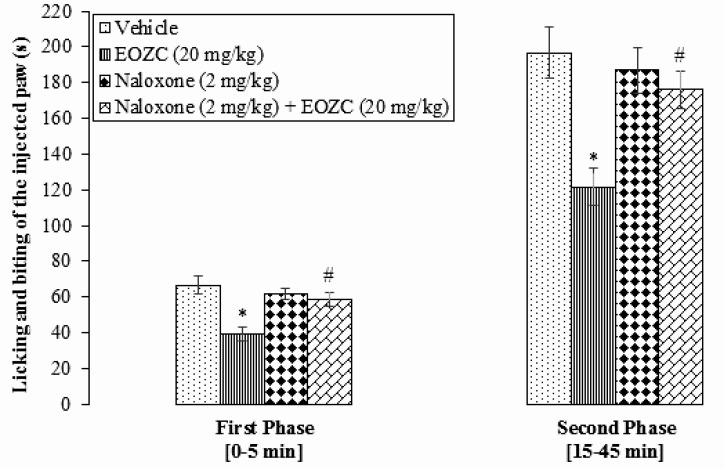
Effect of pretreatment with naloxone on the antinociceptive activity induced by administration of EOZC in formalin pain response in rats Data are expressed as Mean±SEM (n=8). ^*^P<0.05: Compared with vehicle treated group. ^#^P<0.05: Compared with EOZC (20 mg/kg) the test group. EOZC: Essential Oil of *Ziziphora Clinopodioides*.

Based on the findings, naloxone (2 mg/kg) alone had no significant effects on both phases of formalin test (P>0.05). In addition, pretreatment with naloxone (2 mg/kg) significantly reversed antinociception by EOZC (20 mg/kg) in the first (58.50±3.77 s) and second (176.00±10.25 s) phases of formalin test (P<0.05) (Figure 3).

## 4. Discussion

The present study investigated the antinociceptive effect of EOZC and possible involvement of opioidergic system on EOZC-induced antinociception in male rats using formalin test. Herbs and plants are widely used in traditional medicine to treat numerous illnesses due to their potentially positive effects (Sofiabadi et al., 2014). Numerous experiments have explored analgesic effects of medicinal plants (Riedel, Marrassini, Anesini, & Gorzalczany, 2015).

To our knowledge, this is the first report on the interaction of antinociceptive effect of the EOZC and opioidergic system by the formalin test in rats. Two phases of pain were evoked by formalin injection into the hind paw of the animals. Each phase of formalin test has different mechanisms of nociception. The first phase consists of neurogenic nociception, by direct stimulation of nociceptors (via C fibers) to the dorsal horn of the spinal cord after substance P is secreted and acts as a neurotransmitter. The second phase consists of inflammatory-induced pain because of release of various inflammatory mediators such as serotonin, histamine, bradykinin, Prostaglandins (PGs) and excitatory amino acids from the tissue damaged by formalin (Akbari, Mirzaei, & Shahabi Majd, 2013; Abbott, Franklin, & Westbrook, 1995).

Formalin test is a suitable method for generating and quantifying chemical pain in the rat model. Formalin test has long been used as a well-characterized method to evaluate antinociceptive and anti-inflammatory properties of new substances, as novel drugs continue to be developed from plants (Vissers, Hoffmann, Geenen, Biermans, & Meert, 2003; Dubuisson & Dennis, 1977).

Several studies suggest that the centrally acting analgesic drugs like opioids can inhibit both phases of formalin test (Shibata, Ohkubo, Takahashi, & Inoki, 1989), while peripherally acting drugs exert an inhibition only on the second phase of formalin test (Elisabetsky, Amador, Albuquerque, Nunes, & Carvalho Ado, 1995). The IP injection of EOZC revealed a dose-dependent antinociceptive effect on both phases of formalin-induced nociception in rats. Considering the effectiveness of EOZC in suppression of paw licking time in both phases of the formalin test, it seems that the analgesic activity of Z. clinopodioides is mediated by both peripheral and central antinociceptive mechanisms. Based on the GC-MS results, EOZC contained high concentrations of phenolic compounds including carvacrol (65.22%), thymol (19.51%), pcymene (4.86%) and γ-terpinene (4.63%). Several studies have reported the chemical composition of EOZC (Aghajani et al., 2008; Behravan et al., 2007; Ozturk & Ercisli, 2007).

It has been shown that flavonoids and polyphenolic compounds possess a great variety of pharmacological properties including antioxidant activity (Altiok, Altiok, & Tihminlioglu, 2010), immunomodulatory activity (Lima et al., 2012), inhibition of histamine release from mast cells (Amresh, Reddy, Rao, & Singh, 2007a), and suppression of prostaglandin synthesis (Amresh, Zeashan, Rao, & Singh, 2007b). Prior investigations suggested an antinociceptive activity for carvacrol (Guimarães et al., 2010), thymol (Beer, Lukanov, & Sagorchev, 2007), p-cymene (De Sousa, 2011), and γ-terpinene (Hajhashemi, Sajjadi, & Zomorodkia, 2011) in the model of formalin-induced licking. Furthermore, it is reported that thymol partially blocks voltage-operated Na+channels and directly activates Cl- currents via GABAA receptors (Haeseler et al., 2002; Mohammadi et al., 2001).

It was also expressed that thymol reversibly inhibited pros-taglandin synthesis, probably related to the analgesic effect of thymol in endodontic therapy (Sarmento-Neto, do Nascimento, Felipe, & de Sousa, 2016). There is also evidence that the antinociceptive effects of carvacrol are partly related to antioxidant activity and its scavenging activity on NO and other Reactive Oxygen Species (ROS) (Guimarães et al., 2010). Accordingly, based on the above-mentioned findings and given that these 4 compounds are among the predominant components of EOZC, it can be concluded that the anti-nociceptive property of EOZC might be at least in part due to the presence of these chemical compounds.

Morphine was recognized as an effective inhibitor of both phases of formalin pain. Morphine and other opioid analgesics are used for alleviating pain. The opioidergic system consists of 3 receptors including μ, δ and κ which are located in the central nervous system and throughout the peripheral tissues (Trescot, Datta, Lee, & Hansen, 2008). Studies report that the endogenous opioidergic system and its receptors take part in many functions, e.g. behavior, pain and analgesia, stress, tolerance and dependence, learning and memory, alcohol and substance abuse, respiratory control, locomotion, seizures, neurological disorders and neuroendocrine physiology (Bodnar, 2016).

Naloxone (an opioid receptor antagonist) was evaluated in the formalin test to explore the effect of endogenous opioidergic system on the antinociception mechanism exerted by EOZC. Naloxone is a competitive antagonist of μ, δ and κ receptors, with a high affinity for the μ receptor (Trescot et al., 2008). Our findings revealed that the antinociception caused by the EOZC was significantly attenuated by the pre-treatment of rats with naloxone (2 mg/kg), thus it reverses the analgesic activity of EOZC, to some extent.

The obtained results suggest that the constituents in the EOZC may act through opioidergic pathway to produce antinociceptive activity. However, further investigation is required to elucidate the underlying cellular and molecular signaling pathways.

## Ethical Considerations

### Compliance with ethical guidelines

Animal experiments used in this study were approved by the Animal Ethics Committee of Razi University and followed with the Guidelines for the Care and Use of Laboratory Animals in Research (Ethics code: 396-2-012).
